# Diagnostic Methods Used in Detecting Poliomyelitis in Paleopathological Research—A Narrative Review

**DOI:** 10.3390/v18091047

**Published:** 2026-09-21

**Authors:** Julia Gąsiorowska, Maciej Mateusz Hanypsiak, Natalia Jendrzejaczyk, Artur Ams, Jan Rafał Falana, Karol Zagórski, Katarzyna Rekiel, Kinga Brawańska-Maśluch, Pola Nowacka, Wiktoria Żabińska, Grzegorz Mikita, Piotr Paweł Chmielewski, Krzysztof Data, Paul Mozdziak, Bartosz Kempisty, Paweł Dąbrowski

**Affiliations:** 1 Vertex Students’ Scientific Club, Division of Anatomy, Department of Human Morphology and Embryology, Wroclaw Medical University, Ul. Chalubinskiego 6a, 50-368 Wroclaw, Poland; 2 Department of Pediatrics, Endocrinology, Diabetology and Metabolic Diseases, Wroclaw Medical University, Ul. Chalubinskiego 2a, 50-368 Wroclaw, Poland; 3 Division of Anatomy, Department of Human Morphology and Embryology, Wroclaw Medical University, Ul. Chalubinskiego 6a, 50-368 Wroclaw, Poland; 4 Prestage Department of Poultry Science, North Carolina State University, Raleigh, NC 27695, USA; 5 Physiology Graduate Faculty, North Carolina State University, Raleigh, NC 27613, USA

**Keywords:** poliomyelitis, paleopathology, anthropology, differential diagnosis

## Abstract

Objective: This review aims to describe the macroscopic methods used to detect poliomyelitis in human skeletal remains and to highlight the limitations of current diagnostic frameworks. Materials: The reviewed materials consist of published paleopathological literature and case studies analyzing human skeletal remains for evidence of the disease. Methods: We evaluated existing studies that focus on the macroscopic analysis of secondary skeletal alterations caused by paralysis, such as unilateral limb atrophy, bone shortening, pronounced asymmetry, and compensatory hypertrophy. Results: Current diagnostic frameworks rely almost exclusively on macroscopic markers; notably, none of the reviewed studies utilized molecular or genetic techniques. Consequently, definitively confirmed cases of ancient poliomyelitis remain exceptionally rare. Conclusions: The paleopathological diagnosis of poliomyelitis currently suffers from a substantial methodological gap due to an overreliance on morphological assessments. Transcending these traditional techniques is essential to definitively confirm ancient cases. Significance: Analyzing skeletal evidence provides crucial insights into how this debilitating viral infection afflicted human populations in antiquity. It also underscores the importance of rigorous differential diagnosis to distinguish poliomyelitis from other paralytic etiologies, such as cerebral palsy.

## 1. Introduction

Poliomyelitis is a severe and frequently debilitating infectious disease caused by the poliovirus, a member of the Enterovirus genus within the Picornaviridae family of small, positive-strand RNA viruses. Following ingestion, the poliovirus replicates in the oropharynx and gastrointestinal tract, enters the bloodstream (viremia), and, in a small proportion of cases, disseminates to the central nervous system, where it selectively infects and destroys motor neurons in the spinal cord and brainstem, resulting in paralysis [1] Infection with the poliovirus is frequently asymptomatic; approximately 70% of infected individuals present no clinical signs, facilitating the silent transmission of the virus within populations. Among symptomatic individuals, many experience a mild, nonspecific viral illness characterized by fever, fatigue, headache, malaise, myalgia, nuchal or spinal rigidity, and gastrointestinal discomfort lasting several days. A markedly smaller fraction of infections progress to aseptic meningitis (meningeal inflammation without paralysis) or the classical paralytic manifestation of poliomyelitis, wherein the virus induces acute flaccid paralysis of the limbs and, rarely, the respiratory musculature or bulbar palsy [2]. Paralytic poliomyelitis typically occurs in fewer than 1% of infections but can culminate in asymmetrical limb weakness, permanent disability, and even mortality if respiratory muscles are compromised. Survivors of paralytic poliomyelitis may also develop post-polio syndrome (PPS) decades after the acute infection. Characterized by the new onset of muscle weakness, fatigue, and pain, PPS constitutes a long-term sequela of the initial neuronal damage.

While the disease has caused paralysis for millennia—with evidence of withered limbs dating back to ancient Egyptian stelae [3]—widespread, devastating epidemics did not emerge until the late 19th century. Typically, it affected children up to 4 years old, but in the 20th century, there was a demographic shift as the age of affected patients rose to 5–9 years [4]. Widespread vaccination programs have drastically curtailed the global incidence of wild poliovirus infection and remain the cornerstone of worldwide eradication efforts [5].

**Figure 1:**
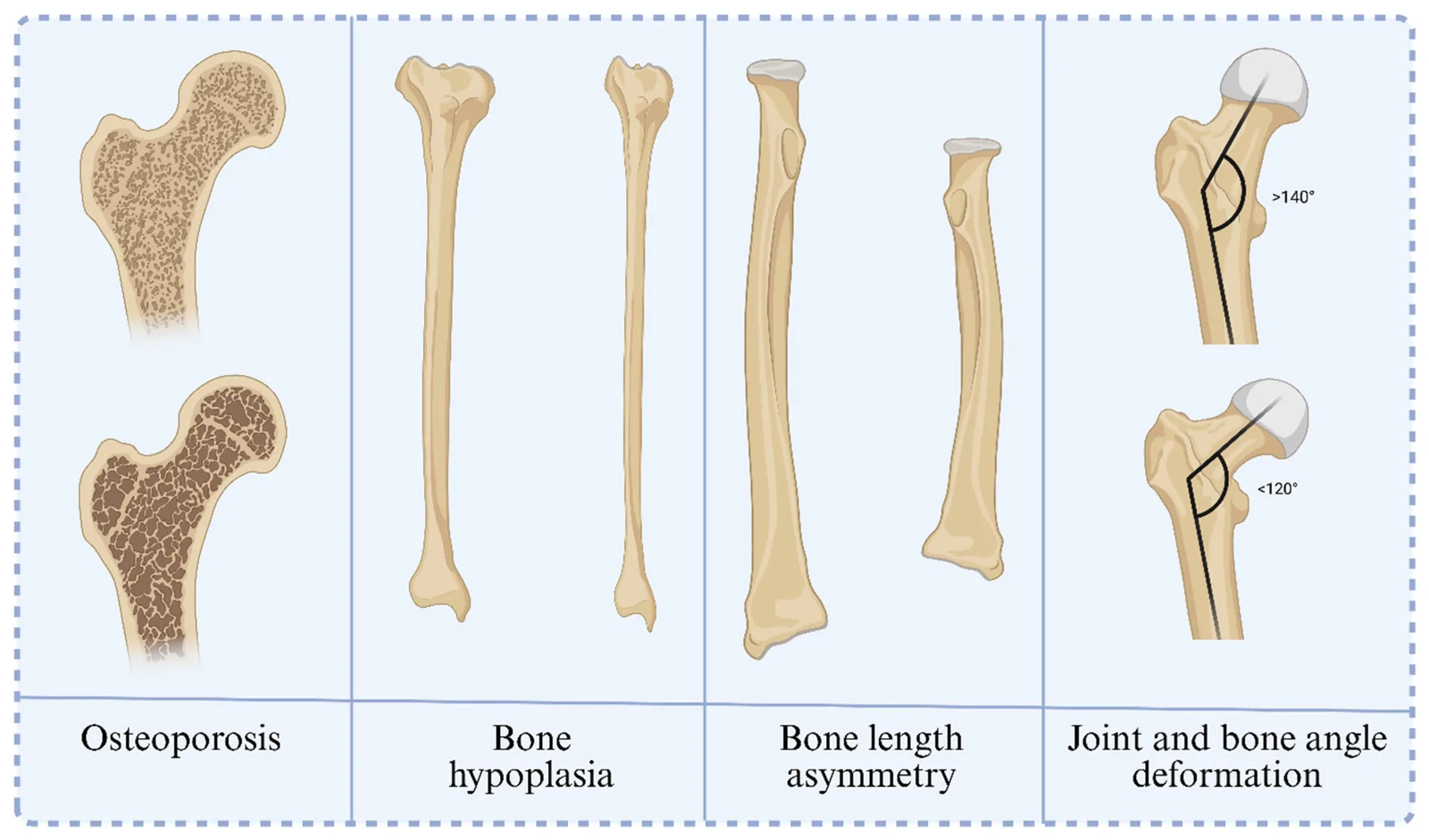
Osteological poliomyelitis markers. Normal bones above or on the left, pathological below or on the right.

The aim of this review is to describe the macroscopic methods used to detect poliomyelitis in human skeletal remains. In paleopathological research, poliomyelitis represents a condition of significant interest. Although direct evidence in ancient skeletons is challenging to ascertain, the virus’s neuromuscular effects can leave discernible signatures. Osteological evidence suggests that the disease may have circulated within human populations long before the advent of modern epidemiological records, with skeletal deformities indicative of paralytic lesions having been attributed to poliomyelitis in archaeological contexts [6]. Valuable insights into the historical trajectory and contemporary relevance of this disease can be derived from paleopathological investigations. However, such studies necessitate robust diagnostic methodologies capable of differentiating these specific markers within human remains. Elucidating the pathological burdens of past generations yields critical demographic data and contextualizes contemporary health challenges. Poliomyelitis poses a substantial diagnostic challenge because its skeletal markers, such as limb atrophy, asymmetry, and secondary biomechanical alterations, as shown in Figure 1, are frequently nonspecific and can be readily conflated with other neuromuscular or paralytic etiologies [7]. Diagnostic protocols established in clinical settings cannot be directly transposed to osteoarchaeological contexts, where remains are frequently fragmentary, poorly preserved, and devoid of corroborating medical histories. Consequently, differential diagnosis is paramount for accurate interpretation [8]. Despite these limitations, documented cases within the paleopathological literature have demonstrated how macroscopic morphological analysis, occasionally augmented by the assessment of entheseal changes and radiological imaging, can facilitate the identification of poliomyelitis in human remains [9].

## 2. Material and Methods

A literature review was conducted between 20 and 28 December 2025, using PubMed, Embase, Web of Science, and Google Scholar. We developed the following search terms:

1. poliomyelitis AND differentiation AND anthropology;
2. poliomyelitis AND molecular AND anthropology;
3. poliomyelitis AND markers AND anthropology;
4. poliomyelitis AND imaging AND anthropology;
5. poliomyelitis AND diagnostics AND anthropology.

Although the main focus of this study is paleopathology, the term “anthropology” was deliberately used in the search query. This approach was adopted because the diagnostic markers of poliomyelitis (e.g., long bone asymmetry, localized atrophy) are fundamentally assessed using anthropometric and osteological methods, which are frequently indexed under broader anthropological disciplines rather than strictly paleopathological ones.

Following the initial review, 55 articles were identified. After the removal of duplicates, 30 articles remained. Ultimately, we included 14 articles that fulfilled the following criteria:

1. The record was an original, peer-reviewed, and published study.

2. The full text was available.

3. The full text was in English.

4. The full text was relevant to the topic of our review: what research methods are used by anthropologists and archaeologists to diagnose poliomyelitis in osseous human remains.

## 3. Results

### 3.1. Current Status of Research

The number of definitively identified cases of ancient poliomyelitis remains exceedingly low because of the difficulty in distinguishing it from other pathological conditions. Most interpretations are inherently tentative, contingent upon the development of more refined approaches, including biomolecular techniques capable of substantiating or refuting preliminary diagnoses. Table 1 presents the gathered articles, which consider poliomyelitis as a more or less probable diagnosis. The data presented were obtained through the macroscopic analysis of osseous material. Additionally, the studies by Berner et al. (2021), Gómez-González et al. (2024), Isidro and Rodriguez (2004), Martin and Potts (2012), and Kozakaitė (2022) [7,8,9,10,11] also utilized X-ray imaging. The period of interest spans from antiquity to the early 20th century. Later cases generally do not require paleopathological diagnosis, as they are well documented in archival medical records.

### 3.2. Osteological Manifestations of Poliomyelitis

Prolonged muscular paralysis is the primary source of osteological changes in poliomyelitis, causing secondary alterations rather than the direct, pathogen-induced bone destruction characteristic of infectious diseases like syphilis or leprosy [21]. Impairment of the peripheral nervous system leads to muscular atrophy, which correspondingly affects the bones. Because these modifications stem indirectly from muscle paralysis instead of direct erosion by the pathogen, they remain nonspecific. This indirect origin fundamentally differentiates poliomyelitis from other infectious conditions and complicates an unambiguous diagnostic assessment. The primary skeletal manifestation of a prior poliomyelitis infection is typically the thinning (gracilization) and longitudinal shortening of the bones within the paralyzed limb [8]. This bone presentation is frequently accompanied by other skeletal deformities, including spinal scoliosis and a femoral deformation characterized by an excessive widening of the femoral neck-shaft angle (coxa valga). Furthermore, several researchers have extended their analyses beyond the paralyzed limbs to evaluate secondary changes in the unaffected extremities. Paralysis in one region of the body significantly elevates the mechanical stress on the unaffected limbs. This sustained mechanical overloading results in cortical hypertrophy and more pronounced musculoskeletal attachment sites (entheseal changes) [14,20].

Several primary osteological indicators are considered suggestive of a prior poliomyelitis infection. These include unilateral longitudinal shortening and the gracilization of the long bones, which are frequently observed in conjunction with disuse osteoporosis within the affected, paralyzed limb.

Beyond these primary markers, researchers must also meticulously evaluate secondary biomechanical adaptations. These secondary changes, such as scoliosis or degenerative joint disease, typically arise as a direct result of the compensatory mechanical overloading placed upon the unaffected side of the individual’s body during locomotion [20].

Determining the age of onset is another critical variable in the diagnostic process. An infection contracted during the developmental years typically inhibits longitudinal bone growth at the epiphyseal plates, leading to significant limb length discrepancies. In contrast, infections occurring in adulthood predominantly precipitate muscular atrophy and subsequent cortical bone loss without resulting in concurrent longitudinal shortening of the limbs [14].

A substantial number of cases described in the existing bioarcheological literature remain categorized merely as ‘probable’ or ‘possible’ diagnoses because of the diagnostic ambiguities observed in the specimens. The frequent absence of definitive, pathognomonic evidence—markers uniquely characteristic of polio—continues to impede the unequivocal confirmation of the disease in the archaeological record [14].

### 3.3. Differential Diagnosis of Poliomyelitis

Poliomyelitis is typically characterized by asymmetric, predominantly unilateral paralysis, which primarily affects the lower extremities [18]. While the poliovirus does not directly infiltrate osseous tissue, the consequent muscular atrophy precipitates significant secondary skeletal remodeling. This manifests as cortical thinning, diaphyseal narrowing (gracilization), and profound osteopenia.

Furthermore, compensatory hypertrophy within the unaffected limb frequently serves as a biomechanical indicator of protracted survival following the onset of the condition. Nevertheless, the formulation of such precise conclusions necessitates the preservation of substantial skeletal elements, which encompass the small bones of the hands and feet, as well as major joints for comparative baselines [10].

The most probable alternative diagnoses in poliomyelitis research are cerebral palsy, leprosy, idiopathic clubfoot, and cardiovascular incidents leading to paralysis [10,18]. (See Table 2).

A fundamental element of the diagnostic procedure involves the differentiation of poliomyelitis from alternative paralytic or neuromuscular disorders. Although cerebral palsy represents a primary candidate for differential diagnosis—given its comparable absence of direct skeletal infection—the diagnostic scope must also be broadened to encompass idiopathic clubfoot, peripheral nerve trauma, and the neurotrophic sequelae of leprosy. The distinction between pathologies is predicated upon a multifactorial analysis concerning the extent of alterations, the identification of affected limbs, and an assessment of pathological symmetry. The presence of related medical conditions or localized muscle wasting might point to physical injury or spinal tuberculosis. Conversely, poliomyelitis is the most probable diagnosis when extreme wasting affects one or two limbs, provided there is no concurrent joint infection or arthritis [22].

## 4. Potential Research Methodology and Techniques

### 4.1. Microscopical Analysis

Highly precise diagnostic conclusions are fundamentally contingent upon the preservation of extensive skeletal assemblages. Both pathological specimens and unaffected elements, which function as indispensable comparative baselines, are necessary to reach accurate diagnostic conclusions. Consequently, the frequent scarcity or fragmentation of such material represents a formidable methodological challenge within the field of paleopathological research [9]. Meanwhile, histological investigations conducted on the same specimen revealed a diminished osteon count, suggesting significant alterations in the bone’s internal structure.

**Figure 2:**
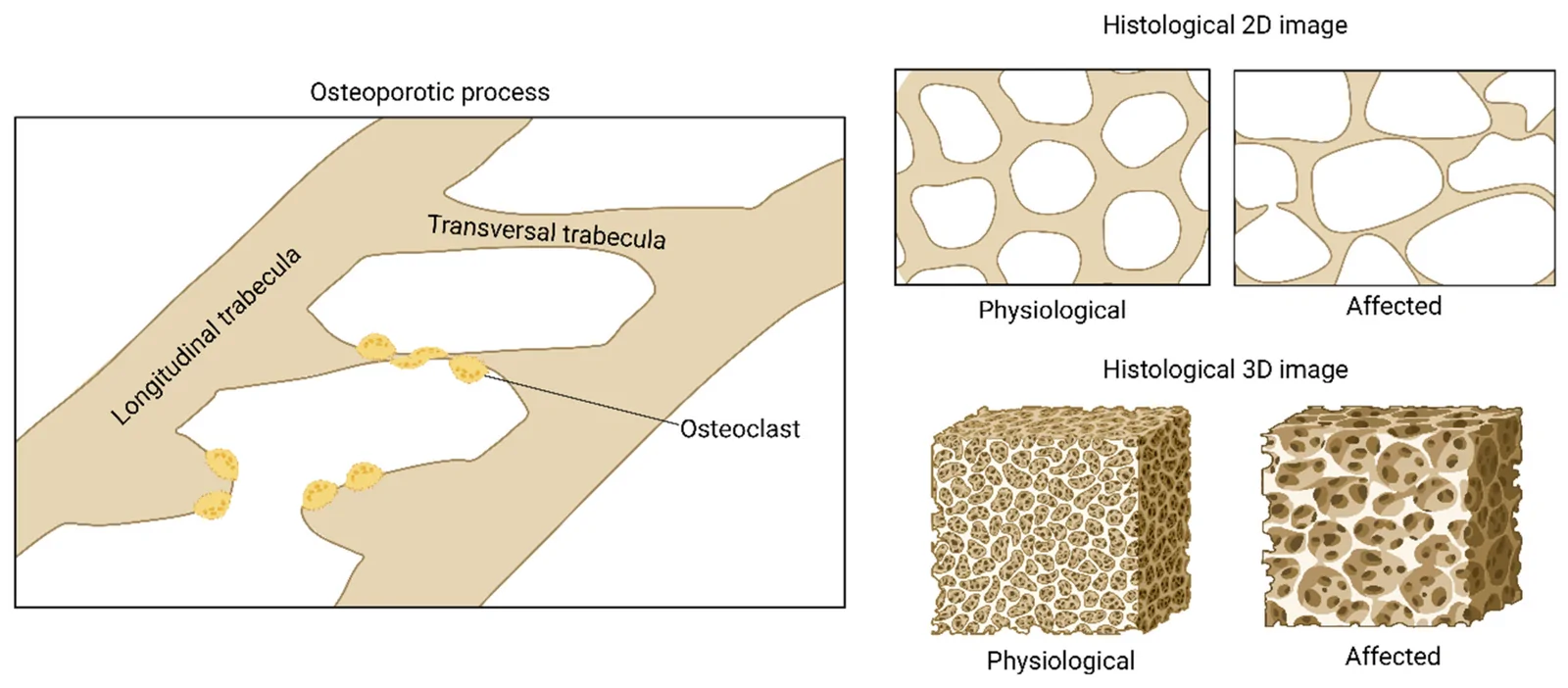
Histological representation of osteoporotic trabecular resorption.

Paleohistological analysis of skeletal remains affected by poliomyelitis may reveal profound secondary microstructural changes caused by neurogenic osteoporosis resulting from a lack of physical activity. Microscopic examination typically reveals significant thinning of the cortical bone, accompanied by increased intramedullary porosity, characterized by enlarged Haversian canals and irregular resorptive spaces resulting from accelerated intramedullary resorption. Furthermore, the trabecular network shows significant weakening and loss of structural integrity, as shown in Figure 2. In cases where the disease occurred in childhood, a marked absence or limitation of secondary osteon remodeling under polarized light indicates a halt in mechanically induced bone maturation, which constitutes the characteristic histological profile of long-term muscle paralysis [23,24]. However, such changes are not pathognomonic for poliomyelitis, but together with macroscopic analysis they can strengthen the diagnosis.

### 4.2. Radiological Approach

Radiological examination of skeletal remains from an individual with a history of poliomyelitis typically reveals pronounced structural modifications driven by chronic muscle atrophy and altered biomechanical loading. As observed in microscopic research, cortical thinning and a reduction in trabecular density may be seen, consistent with severe disuse osteoporosis. The long bones of the affected limbs often exhibit significant hypoplasia, characterized by diminished diaphyseal diameters and shortened lengths, which manifest as conspicuous limb length discrepancies. Furthermore, the absence of normal muscular tensile forces results in underdeveloped entheses [23,24]. X-ray imaging is employed in poliomyelitis research, but it appears that its wider use could prove valuable in uncertain cases. It has to be noted, that like microscopic approaches, radiology can improve the diagnosis, but are not specific enough to prove diagnosis of poliomyelitis by itself.

### 4.3. Archaeogenetics

Despite the availability of advanced diagnostic tools in other biological disciplines, there remains a conspicuous absence of contemporary molecular techniques. Specifically, the polymerase chain reaction (PCR) has not been used to diagnose poliomyelitis in ancient skeletons, even though PCR-based diagnostic protocols for poliomyelitis are routinely employed in modern clinical settings [25]. The specific viral etiology of certain diseases poses unique difficulties, particularly regarding the rapid degradation of the pathogen’s RNA and the low number of virions in affected bones. These factors may introduce significant technical constraints that impede successful molecular recovery. During the PCR procedure, stable RNA or DNA is multiplied and marked, so the primary requirement is the presence of untainted genetic material. As the poliovirus is an RNA virus, RNA preservation is extremely poor over archaeological timescales. To survive to the present day, it would have to resist environmental RNases, withstand hydrolysis, avoid contamination, and persist in bone material. Even if it were to survive, it would likely remain undetected because bone is not a primary site of viral replication or persistence. The virus predominantly infects the gastrointestinal tract and associated lymphoid tissues, with only a transient presence in the bloodstream during the viremic phase of infection.

**Figure 3:**
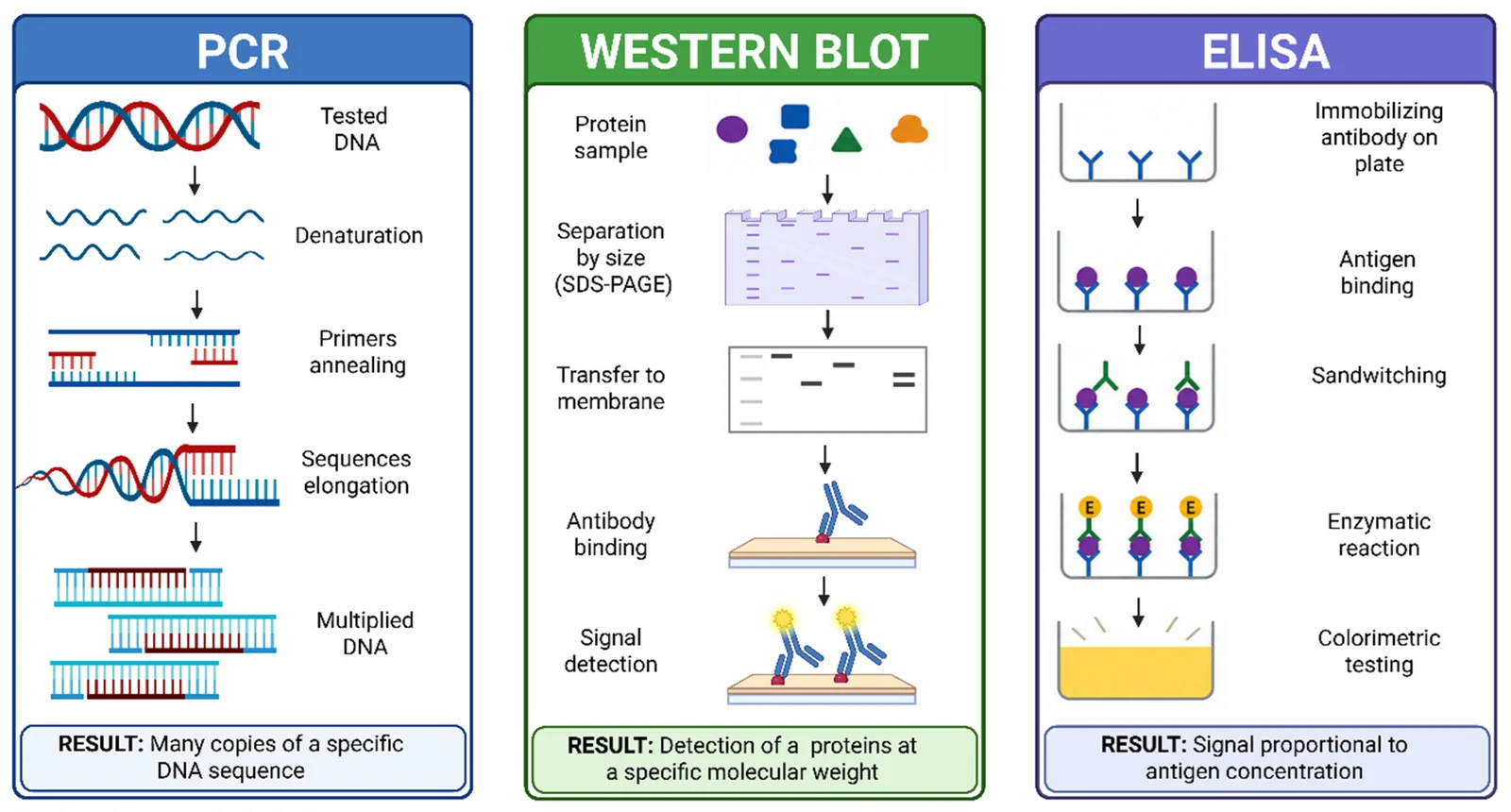
Schematical comparison of polymerase chain reaction, Western blot and ELISA assays.

Consequently, the modern molecular diagnosis of poliomyelitis relies on specimens such as stool, throat swabs, blood, and, less commonly, cerebrospinal fluid, where viral RNA is more likely to be detected. Furthermore, the poliovirus rarely accumulates in tissues that are typically preserved in the archaeological record. Another challenge is the high cost of molecular research, which is significantly more expensive than a classic macroscopic examination. However, further investment into molecular diagnostic research could yield significant and measurable benefits, ultimately advancing the analytical capabilities of the discipline, considering that the application of genetic techniques such as ELISA, Western blot (WB), and PCR has already achieved substantial success within paleopathology, particularly in the molecular identification of chronic bacterial infections such as tuberculosis and leprosy [26,27,28,29]. For these diseases, the analysis of genetic material—which is relatively often preserved due to the biochemistry of the mycobacterial cell wall—yields good results; however, WB and ELISA are also effective in detecting substances specific to these microorganisms, such as mycolic acids. In this way, they provide very strong, nearly conclusive evidence confirming an infection, although the absence of such evidence cannot be used as proof against it. When combined with macroscopic examination, these methods allow for a diagnosis to be made with a high degree of certainty. Characteristics of ELISA, WB, and PCR are shown in Figure 3. Despite the destructive nature of these procedures, they are considered valuable because of their significantly higher sensitivity and specificity [30].

Notwithstanding these hurdles, the inherently low specificity of macroscopic skeletal alterations necessitates a shift toward more rigorous methods. Perhaps research on specific materials, such as natural mummies preserved in ice caps or deserts, might be a good starting point. Preserved soft tissues could be a source of poliomyelitis molecules, which might later serve as a reference in less well-preserved cases.

## 5. Conclusions

The identification of poliomyelitis within the context of paleopathological investigation represents a formidable diagnostic challenge for researchers, primarily because the direct detection of the viral pathogen within ancient human remains is exceptionally difficult to achieve. The fundamental obstacle complicating these studies is the inherently nonspecific nature of the associated skeletal alterations. Diagnostic bone manifestations, which typically include marked asymmetry, pronounced limb atrophy, and various pedal deformities, are not unique to poliomyelitis and may be readily misidentified as or conflated with other neuromuscular etiologies.

Furthermore, established clinical diagnostic protocols used in modern medicine are not directly applicable to the field of osteoarcheology. Researchers must frequently contend with incomplete or fragmentary skeletal assemblages and are hindered by a total absence of corroborating medical histories or soft-tissue evidence. Consequently, the implementation of a rigorous and exhaustive differential diagnosis is considered imperative. This diagnostic process is necessary to systematically exclude alternative conditions that present with similar osteological profiles, such as cerebral palsy, cerebrovascular accidents (stroke), congenital dysplasia, and Rasmussen’s encephalitis.

The total number of reliably identified cases of ancient poliomyelitis remains exceedingly low on a global scale because of the difficulty in definitive diagnosis. Any interpretation of suspected cases demands a meticulous, multifaceted analysis of the entire individual skeleton to ensure accuracy. Therefore, it is highly justifiable to advocate for, and support, further research directed toward the development of novel diagnostic methodologies. Such advancements should aim to integrate diverse scientific disciplines. By synthesizing these approaches, the scientific community may improve the precision in identifying the disease.

## Figures and Tables

**Table 1:** Reported cases of poliomyelitis in archaeological literature. All of them included one skeleton, except Gómez-González et al. (2024) [9], featuring four, and Novak et al. (2014) [12], featuring two.

Article	Region	Historical Period	Relevant Skeletal Pathologies
[7]	Austria	II–V century	Scoliosis, atrophy of the right humerus and both lower limbs with simultaneous hypertrophy of the left arm. In X–ray examination mild thinning of cortex in atrophied bones. Reduced number of osteons in microscopic investigation. Faint or none expression of entheses on affected bones, except of left arm, where especially on humerus there was a distinct expression
[8]	Lithuania	XVI–XVII century	Right-side scoliosis, edge–shaping of lumbar vertebrae, gracilization of lower extremities long bones and left upper extremity
[9]	Spain	XX century	Scoliosis, coxa valga, significant asymmetry and atrophy of the left lower limb long bones, deformation and tilting of the pelvis, torsion of the left tibia, clubfoot
[13]	Egypt	XX–XVIII century BC	Gracilization of right humerus, scapula, tibia and fibula. Atrophy of right foot bones
[14]	USA	XIX century	Scoliosis, degenerative disc disease and osteophytosis, gracilization and atrophy of left lower extremity long bones, clubfoot. On a contrary, upper limbs were not atrophied and relatively robust.
[15]	Great Britain	II–IV century	Scoliosis, left humerus, femur and tibia atrophied and gracile. Cranium and pelvis asymmetric, smaller on the left side. Enamel hipoplasia on first mandibular molars
[16]	Serbia	XII–XIII century	Coxa valga
[10]	Canary Islands	XII–XIII century	Osteochondritis of trochlea, osteophytes of subtalar joint, clubfoot
[17]	Portugal	XV century	Scoliosis, lesions of cervical, thoracic and lumbar vertebrae, ribs and acromio–clavicular joints, coxa valga on the right side, atrophic acetabulum, thibia, fibula and patella
[18]	Sudan	VII–XIII century	Atrophy and gracilization of left lower limb long bones
[12]	Croatia	XII–XV century	Scoliosis, coxa valga, shortening of lower limbs long bones
[19]	Spain	Unknown	Gracilization of femur
[11]	United Arab Emirates	Bronze Age	Unilateral femoral gracilization, foot deformities, pelvic abnormalities suggesting chronic sitting
[20]	United Arab Emirates	Bronze Age	Gracilization of lower limb bones, foot deformities. Hypertrophy of the humeri, radii, and ulnae. High expression of humeral, radial, ulnar and clavicular entheses.

**Table 2:** Differential diagnosis of paralytic and neuromuscular disorders in paleopathological analysis.

Condition	Manifestation	Symmetry and Distribution	Key Diagnostic Differences
Poliomyelitis	Gracilization and longitudinal shortening of long bones. Secondary deformities such as coxa valga, scoliosis, and talipes equinovarus	Characteristically asymmetric and predominantly unilateral, primarily affecting the lower extremities	Absence of direct osteological destruction; diagnosis relies on secondary alterations from muscular paralysis
Cerebral palsy	Disuse atrophy and secondary biomechanical alterations similar to those of poliomyelitis	Often involves different limb combinations; distribution depends on the specific type of palsy	Frequently presents with a more varied or systemic distribution of skeletal markers compared to the typical unilateral focus of polio
Leprosy	Neurotrophic sequelae, often involving the resorption of phalanges and rhinomaxillary changes	Can be bilateral or asymmetric depending on the clinical stage	Distinguished by direct pathogen-induced bone erosion and characteristic destruction of the hands, feet, and face
Idiopathic clubfoot	Isolated deformity of the foot (e.g., talipes equinovarus) without systemic limb involvement	Can be unilateral or bilateral	Lacks the extensive long-bone gracilization and longitudinal shortening characteristic of early-onset polio
Cerebrovascular incident (stroke)	Hemiplegic atrophy and secondary degenerative changes resulting from prolonged paralysis	Unilateral	Primarily affects adults; lacks the longitudinal bone growth inhibition seen in childhood-onset polio

## Data Availability

All data supporting this work are from previously published studies, which are cited in the reference list.
